# Trastuzumab therapy *vs* tetracycline controlled ERBB2 downregulation: influence on tumour development in an ERBB2-dependent mouse tumour model

**DOI:** 10.1038/sj.bjc.6604318

**Published:** 2008-04-29

**Authors:** M Hermes, W Schormann, M Brulport, K Uhlemann, F Lupatsch, L C Horn, A Schumann, C Allgaier, M Weishaupt, K Engeland, G A Müller, J Mössner, A Bauer, I B Schiffer, S Gebhard, M Schmidt, E Lausch, D Prawitt, C Wilhelm, J G Hengstler

**Affiliations:** 1 Leibniz Research Centre for Working Environment and Human Factors, University of Dortmund, Ardeystraße 67, Dortmund D-44139, Germany; 2 Institute of Legal Medicine/Rudolph-Boehm-Institute of Pharmacology and Toxicology, Leipzig D-04103, Germany; 3 Institute of Pathology, Division of Gynaecopathology, University of Leipzig, Leipzig D-04103, Germany; 4 Institute of Biology I, Division of Plantphysiology, University of Leipzig, Leipzig D-04103, Germany; 5 Department of Obstetrics and Gynaecology, Medical School, University of Leipzig, Semmelweisstr. 14, Leipzig D-04103, Germany; 6 Department of Internal Medicine II, Max Burger Research Centre, University of Leipzig, Johannisallee 30, Leipzig D-04107, Germany; 7 Department of Obstetrics & Gynaecology, Medical School, University of Mainz, Mainz D55101, Germany; 8 Medical Genetics and Molecular Medicine, University of Mainz, Mainz 55101, Germany

**Keywords:** breast cancer, trastuzumab, herceptin, response to therapy, resistance, ERBB2, HER2, receptor tyrosine kinase, ERK1/2, Akt, PKB, Ki-67, cytochrome *c* release, tumour development, humanised monoclonal antibody, nude mice

## Abstract

Trastuzumab (Herceptin) has improved therapy of breast cancer. Only patients overexpressing ERBB2 are treated with trastuzumab, whereas its use in tumours without ERBB2 expression is useless. This led to the concept that the subgroup of trastuzumab-sensitive tumours is ‘ERBB2-dependent’, meaning that ERBB2 signalling is indispensable for growth of these tumours. We used a mouse model that allows anhydrotetracycline (ATc)-controlled downregulation of ERBB2 in tumour tissue. ERBB2 mRNA and protein expression were downregulated below detection limit leading to a macroscopically complete tumour remission within 14 days. Tumour remission was accompanied by a strong decrease in proliferation, a moderate increase in apoptosis, as well as dephosphorylation of ERK1/2 and AKT/PKB. These data clearly indicate ERBB2 dependence. Therefore, a high sensitivity to trastuzumab may be suspected. Surprisingly, trastuzumab caused a much weaker effect compared to ATc-induced ERBB2 downregulation, although a decrease in ERBB2 membrane localisation was induced. Only a slight decrease in proliferation and a weak transient increase in apoptosis were observed. Interestingly, tumours responded to trastuzumab by a sharp fivefold increase in phosphorylated AKT/PKB as well as a 3.5- and 5.3-fold increase in AKT1 and AKT2 mRNA levels, respectively. In conclusion, ‘ERBB2 dependence’ is not sufficient to define trastuzumab-responsive tumours. The suboptimal effect of trastuzumab compared to the maximally possible effect induced by ATc demonstrates a high potential for improved ERBB2 blocking therapies.

Since its discovery as a human oncogene, ERBB2 has been intensively investigated as a target for therapeutic intervention (review: (Ullrich and Schlessinger, 1990; Zwick *et al*, 2000)). Among a variety of strategies two have shown the most promising results. One approach utilises small molecule inhibitors targeting the kinase domain (review: (Fischer *et al*, 2003)). The second approach is the generation of antibodies directed against the extracellular domain of ERBB2 (Hudziak *et al*, 1989; Fendly *et al*, 1990; Scott *et al*, 1991). Trastuzumab is a humanised monoclonal antibody that binds to the membrane adjacent cysteine rich part of the extracellular domain of ERBB2 (Cho *et al*, 2003). Several distinct mechanisms are responsible for its antitumour activity: (i) internalisation of ERBB2 from the cell surface (Wartlick *et al*, 2004), (ii) blockade of metalloprotease-induced cleavage of ERBB2 generating a soluble extracellular domain and a kinase active intracellular fragment (Molina *et al*, 2001) and (iii) antibody-dependent cytotoxicity mediated by the Fc portion of trastuzumab (Clynes *et al*, 2000).

Trastuzumab (Herceptin) has significantly improved therapy of breast cancer. In the 1990s trastuzumab was shown to induce tumour remission in patients with metastatic breast cancer after failure of conventional chemotherapy (reviewed by Baselga, 2006). In subsequent studies trastuzumab was found to improve survival when combined with chemotherapy or given sequentially after chemotherapy (Slamon *et al*, 2001; Piccart-Gebhart *et al*, 2005; Romond *et al*, 2005). In large studies combining more than 14 000 patients the addition of trastuzumab to systemic chemotherapy resulted in a 52% reduction of recurrence 4 years after surgery (reviewed by Tsuda, 2006). Trastuzumab clearly enhanced the activity of anthracyclines, taxanes and platinum compounds.

In recent years many scientists have concentrated on the question why some patients respond to trastuzumab whereas others do not (Cappuzzo *et al*, 2006; Chon *et al*, 2006; Menendez *et al*, 2006; Tseng *et al*, 2006). A correlation between the extent of ERBB2 expression determined by immunohistochemistry or FISH and the clinical response has been observed in several studies (Vogel *et al*, 2002; Mass *et al*, 2005; Tsuda, 2006). Women whose breast tumours stain 3+ for ERBB2 (meaning that strong complete membrane staining is seen in more than 10% of tumour cells) are most likely to respond to trastuzumab (Vogel *et al*, 2002; Mass *et al*, 2005) (reviewed by Tokunaga *et al*, 2006). The activity of trastuzumab in breast carcinomas overexpressing ERBB2 has contributed to the concept that certain tumours are ‘oncogene dependent’ (Weinstein, 2002; Baselga, 2006; Hengstler *et al*, 2006). This concept suggests that trastuzumab should be used only for the subset of tumours whose growth depends on the oncogenic signal of ERBB2. In the present study, we made the surprising observation that a ‘ERBB2-dependent’ tumour can be completely resistant to trastuzumab. We used NIH 3T3 cells expressing ERBB2 by the TET-OFF system (Schiffer *et al*, 2003). These cells form rapidly growing tumours in nude mice. Switching-off ERBB2 expression by tetracycline administration causes a rapid and complete tumour remission, demonstrating a high degree of ‘ERBB2 dependence’. In the same tumours trastuzumab caused translocation of ERBB2 from the membrane to the cytoplasm but tumour growth was only slightly delayed. Our study demonstrates that cells can be ERBB2 dependent but nevertheless resistant to trastuzumab.

## MATERIALS AND METHODS

### Cells with ERBB2 expression under control of tTA

NIH3T3 is an immortalised cell line originally derived from mouse embryo fibroblasts and was obtained from ATCC (American Type Culture Collection). Wild type NIH3T3 and its derivatives were grown in Dulbecco's modified Eagle's medium (DMEM, PAN; Aidenbach, Germany) supplemented with 10% fetal bovine serum (tetracycline free, Biochrom AG, Berlin, Germany) and 1% penicillin–streptomycin (PAN). Cells were cultured at 37°C in 5% CO_2_ humidified air (Schiffer *et al*, 2003; Hausherr *et al*, 2006). Conditional expression of ERBB2 was achieved using the TET-OFF system originally described by Gossen and Bujard (Gossen and Bujard, 1992). Briefly, wild type NIH3T3 cells were co-transfected with three vectors (pUHD 15-1, pTBC1 Hygro® and pTBC ERBB2/SEAP) as described by Baasner *et al* (1996) resulting in a cell line termed NIH3T3-HER2. Cotransfection resulted in tetracycline controlled ERBB2 expression: exposure of cells to anhydrotetracycline hydrochloride (ATc) lead to a complete down regulation of ERBB2 (Schiffer *et al*, 2003). Selection for stable transfection was achieved by adding 125 *μ*g ml^−1^ hygromycin B (Sigma-Aldrich, Schnelldorf, Germany) to the cell culture medium. Expansion of NIH3T3-HER2 cells was performed in the presence of hygromycin B. In contrast, all experiments including exposure to ATc were done in the absence of hygromycin B.

### Induction of ERBB2-dependent tumours and determination of tumour growth

NIH3T3-HER2 cells (7 × 10^6^) were subcutaneously injected into the dorsal skin of 3- to 4-weeks-old male nude mice (cd nu−/nu−) (Charles River, Sulzfeld, Germany). Animals were housed under specific pathogen-free conditions. Eight to ten days after injection of the NIH3T3-HER2 cells, small tumours with a mean diameter of 0.5 cm became visible. The tumour diameter was measured using a calibre rule. Maximal and minimal diameters of the tumours were determined. The mean value of the maximal and minimal diameters was defined as the mean diameter. Tumour volume (*V*) was calculated by the formula: *V*=*abb*/2, whereby ‘*a*’ represents the minimal and ‘*b*’ represents the maximal tumour diameter. A complete remission was achieved, when absolutely no tumour volume was macroscopically visible. Anhydrotetracycline was administered to the mice by subcutaneous (s.c.) injection. Experiments reported here were approved by the local Ethical Committee, in accordance with the Declaration of Helsinki and National Institutes of Health guidelines.

### Western blot analysis

#### Preparation of tumour tissue

Mice were killed by cervical dislocation and tumours were isolated, shock frozen in liquid nitrogen-cooled 2-methylbutane and stored at −80°C. To pulverise the tumour tissue a mortar and pestle on dry ice was used. Then the samples were lysed in solubilisation buffer by sonification (10 strokes, three times; Labsonic U, B. Braun Medical AG, Emmenbrücke, Germany) on ice. To remove cell debris, the suspensions were centrifuged at 18 000 **g** for 10 min. The supernatants were collected and protein concentration was estimated by the BCA assay, according to the manufacturer's protocol. The Lyses buffer which was used, contained 25 mM Tris-phosphate, 2 mM EDTA (ethylenediamine tetra-acetic acid), 2 mM DTT (1,4-dithio-threitol), 10% glycerol and 1% Triton-X-100 (pH 8). The solubilisation buffer was supplemented with 1% of a commercially available protease inhibitor cocktail (Sigma) containing 4-(2-aminoethyl)benzenesulphonyl fluoride (AEBSF), pepstatin A, trans-epoxy succinyl-L-leucylamido(4-guanidino)butane (E-64), bestatin, leupeptin and aprotinin.

#### Preparation of mitochondrial and cytosolic extracts for cytochrome *c* detection

Frozen tumour tissue specimens were put into ice-cold homogenising buffer (250 nmol l^−1^ sucrose, 10 mmol l^−1^ HEPES, 1 mmol l^−1^ EDTA, 1 mmol l^−1^ EGTA, 5 mmol l^−1^ DTT, 2 *μ*g ml^−1^ aprotinin and 2 *μ*g ml^−1^ leupeptin). After homogenisation with a Potter-Elvehjem teflon-glass homogeniser (20 strokes), the homogenates were centrifuged at 7000 **g** for 10 min, 4°C. Supernatant was collected after centrifugation at 10 000 **g** for 30 min at 4°C. The supernatants and the pellets were analysed by western blotting. The supernatant represents the cytosolic fraction, whereas the pellet contains the mitochondria.

#### Western blotting

Total cellular protein (50 *μ*g for all analysis) were mixed with sample buffer according to the protocol of Laemmli (25) and resolved on a 10% (HER-2) or on a 12% (phospho akt, akt, phospho p44/42 and p44/42 MAP kinase) SDS–polyacrylamide gel by electrophoresis. Thereafter, proteins were electrotransferred onto Poly Screen PVDF (polyvinylidene-difluoride) Transfer Membranes (NEN® Life Science, Boston, MA, USA). The membranes were blocked with PBST (PBS+0.1% Tween 20) containing 10% Roti®-Block (Roth, Karlsruhe, Germany) for 1 h and then incubated with the anti HER-2/neu for 2 h at room temperature (RT) or the anti cytochrome *c*, phospho akt, akt, phospho p44/42 and p44/42 MAP kinase antibodies overnight at 4°C or as a loading control with the anti *β*-actin antibody for 30 min at RT. After washing, the membranes were incubated with the secondary antibody, horseradish peroxidase conjugated anti-mouse immunoglobulin for 20 min or anti-rabbit immunoglobulin for 60 min at RT. The secondary antibody to detect the cytochrome *c* primary antibody was incubated for 60 min. After final washing, proteins were visualised with a chemiluminescence detection system (Western Lightning™ Chemiluminescense Reagent Plus, PerkinElmer Life Science, Boston, MA, USA) and with subsequent exposure to an Imager System (INTAS, Göttingen, Germany). The expression levels were quantified using GelProAnalyzer software (GelProAnalyzer 4.5 for Windows 2000). For evaluation of apoptosis the ratios of cytosolic and mitochondrial cytochrome *c* were calculated. The MagicMark™ Western Standard from Invitrogen (Invitrogen GmbH, Karlsruhe, Germany) served as an internal protein standard. For repeated staining, individual membranes were stripped with a buffer containing 0.76% Tris-base, 2% SDS and 0.7% 2-mercaptoethanol adjusted with HCl to pH 6.8 for 1 h at 50°C. The SDS–PAGE and the membrane (after the last staining with antibodies) were further stained with Coomassie blue to confirm equal amount of proteins applied in each lane.

#### Origin and dilution of antibodies

The monoclonal antibody against human HER-2/neu (a 185 kDa protein) was obtained from Quartett (Berlin, Germany) and used in a dilution of 1 : 270. The monoclonal antibody against mouse *β*-actin (a 42 kDa protein) (Sigma) was used in a dilution of 1 : 5000. The anti-mouse phosphor-akt antibody (Cell Signaling) was diluted 1 : 200, the anti-mouse akt antibody, the anti-mouse phosphor-p44/42antibody and the anti-mouse phosphor-p44/42antibody were obtained from Cell Signaling and all were diluted 1 : 1000. The secondary antibody, peroxidase-linked anti-rabbit (Cell Signalling) was used in a dilution of 1 : 1000. Peroxidase-linked anti-mouse antibody, obtained from Sigma, was used in a concentration of 1 : 5000 to detect *β*-actin and 1 : 50 000 to detect HER-2. The antibody against cytochrome *c* (BD) was used in a dilution of 1 : 1000 and the secondary antibody anti-mouse 1 : 2000 (Sigma). Primary and secondary antibodies were all diluted in PBS-T containing 10% Roti®-Block.

#### Immunohistochemical detection of Ki-67

In addition to the staining with the anti-human HER-2 antibody, the same protocol was also performed using an anti-mouse Ki-67 antibody (Ki-67 rabbit anti-mouse, Dianova, Hamburg, Germany) diluted 1 : 50 in TBS containing 5% fetal calf serum.

#### Immunohistochemical detection of Her-2/neu

Her-2/neu was analysed immunohistochemically in paraffin sections using the Hercep Test^R^, an FDA-approved assay for identification of tissues overexpressing p185 Her2 (K5205 Dako, Denmark), in accordance with the manufacturer's protocol and scoring guidelines.

#### Evaluation of immunohistochemical slides

Using the anti-mouse Ki-67 antibody the percentage of Ki-67 positive tumour cells was determined in relation to all tumour cells. For this purpose five representative areas with vital tumour cells or tissue were randomly selected. Evaluation was performed using an Olympus microscope (BX-41) with 100-fold magnification. The percentage of Ki-67 (nuclear staining) positive cells was determined independently by two experienced investigators (MH; MW). Mean values for all five areas were calculated. In all cases the values obtained by both investigators differed by less than 10%. ERBB2 membrane localisation was evaluated by an experienced pathologist (LCH) on blinded slides.

## RESULTS

### ERBB2 downregulation causes massive tumour remission

**Figure 1:**
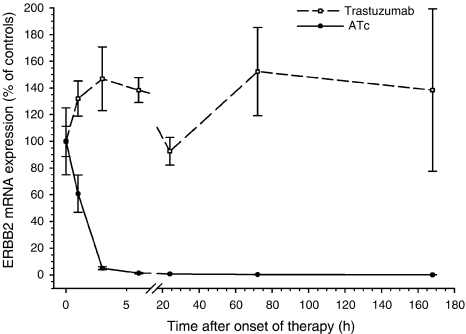
Influence of anhydrotetracycline (ATc) (10 mg kg^−1^, s.c., daily) and trastuzumab (40 mg kg^−1^, ip, daily) on ERBB2 mRNA expression in tumour tissue. Data are mean values and standard errors of 3–5 mice per group. Mean expression level of controls corresponds to 100%.

**Figure 2:**
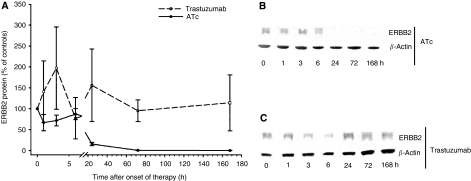
(**A**) Influence of anhydrotetracycline (ATc) and trastuzumab on ERBB2 protein expression levels in tumours of mice; (**B**) Representative immunoblot showing ERBB2 downregulation during ATc therapy; (**C**) Representative immunoblot showing ERBB2 downregulation during trastuzumab therapy. Data are mean values and s.e. of 3–5 mice per group. Mean expression level of controls corresponds to 100%.

**Figure 3:**
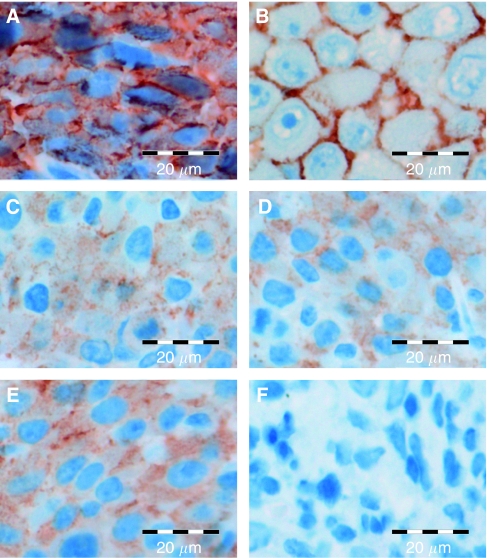
Influence of anhydrotetracycline (ATc) and trastuzumab on membrane localisation of ERBB2. (**A**) Untreated NIH3T3-HER2 tumour; (**B**) positive control; (**C**) NIH3T3-HER2 tumour 3 h after injection of trastuzumab; (**D**) NIH3T3-HER2 tumour treated for 7 days with trastuzumab; (**E**) NIH3T3-HER2 tumour 3 h after injection of ATc; (**F**) NIH3T3-HER2 tumour treated for 7 days with ATc.

**Figure 5 fig5:**
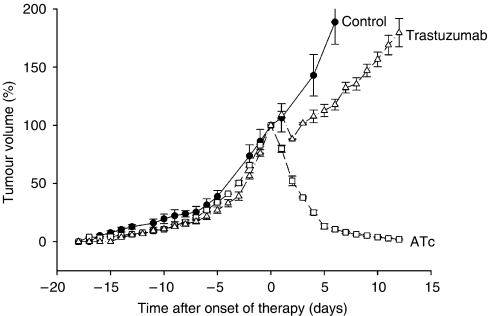
Influence of anhydrotetracycline (ATc) and trastuzumab on tumour development in nude mice. As soon as tumours reached a mean diameter of 1.5–1.8 cm (corresponding to 1.7–2.0 cm^3^) mice received daily injections of anhydrotetracycline (ATc) and trastuzumab (onset of therapy is day 0). Data are mean values and s.e. of 3–5 mice per group. A tumour volume of 100% corresponds to 1.7–2.0 cm^3^.

We used a mouse tumour model that allows tetracycline-controlled expression of ERBB2. This mouse model is based on NIH3T3-HER2 cells that conditionally express ERBB2 by the TET-OFF system. NIH3T3-HER2 cells were injected into the dorsal skin of nude mice to induce subcutaneously growing tumours. Untreated tumours expressed relatively high levels of ERBB2 mRNA (Figure 1) and protein (Figure 2). Immunostaining by the HercepTest showed a strong membrane and cytoplasmic staining (Figure 3A) corresponding to score 3+ of human breast carcinomas (Figure 3B). As soon as the tumours reached a volume of 1.7 cm^3^ mice were either treated with anhydrotetracycline (ATc, daily i.p. injections of 10 mg kg^−1^ for 7 days) or with trastuzumab (daily i.p. injections of 40 mg kg^−1^ for 7 days; see next paragraph). Injection of ATc resulted in downregulation of ERBB2 mRNA expression below detection limit within 24 h (Figure 1). Similarly, immunoblot analyses showed a decrease in ERBB2 expression (Figure 2). After 3 days of ATc exposure ERBB2 protein expression was no longer detectable. ATc induced downregulation of ERBB2 mRNA and protein led to a rapid reduction in tumour volume (Figure 5). Tumour volumes decreased by 20, 48, 62, 75, 87, 89 and 92% after 1, 2, 3, 4, 5, 6 and 7 days of ATc administration, respectively, compared to the tumour volume before therapy. The results show that the tumour cells in our mouse model are highly ERBB2 dependent.

### Trastuzumab decreases ERBB2 membrane localisation but causes only a slight delay in tumour growth

The influence of trastuzumab was tested under similar conditions as described above for ATc. As soon as tumours reached a volume of 1.7 cm^3^, the mice received daily i.p. injections of 40 mg kg^−1^ trastuzumab. In contrast to ATc administration trastuzumab did not cause a decrease in ERBB2 mRNA (Figure 1) or protein (Figure 2C) expression. Nevertheless, the influence of trastuzumab could clearly be detected on a biochemical level by an alteration in subcellular localisation of ERBB2. Before onset of trastuzumab therapy ERBB2 showed a strong membrane and cytoplasmic staining (Figure 3A). Six and 72 h after administration of trastuzumab membrane staining was strongly reduced (Figure 3C and D;). In contrast to ATc, trastuzumab caused only an initial, weak delay of tumour progression (Figure 5). Therefore, despite their dependence on ERBB2, NIH3T3-HER2 tumours are relatively resistant to trastuzumab.

### Trastuzumab increases Akt phosphorylation and mRNA synthesis

**Figure 6 fig6:**
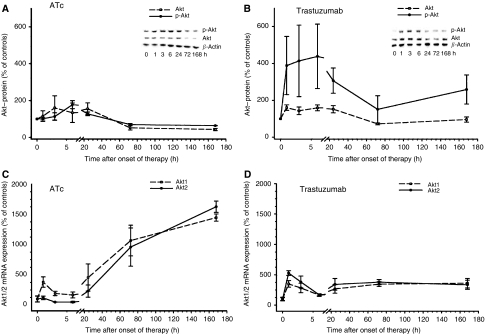
Influence of anhydrotetracycline (ATc) (**A**) and trastuzumab (**B**) on levels of phosphorylated and total Akt. Similarly, the influence of anhydrotetracycline (ATc) (**C**) and trastuzumab (**D**) on Akt1 and 2 mRNA levels was analysed. Data are mean values and s.e. of 3–5 mice per group. Mean expression levels of controls correspond to 100%.

**Figure 7 fig7:**
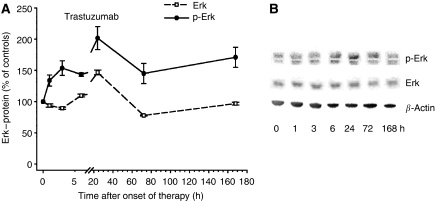
Influence of trastuzumab (**A**) on levels of phosphorylated and total Erk. Representative immunoblot is shown (**B**). Data are mean values and s.e. of 3–5 mice per group. Mean expression level of controls correspond to 100%.

Phosphorylation of Akt is known as an antiapoptotic response to stress (Schiffer *et al*, 2003; Hausherr *et al*, 2006). Therefore, we studied levels of phosphorylated and total Akt in tumour tissue after administration of ATc or trastuzumab. ATc initially (3–6 h after administration) caused a slight approximately 1.8-fold increase in phosphorylated Akt, followed by a sustained decrease after 3 and 7 days of ATc exposure (Figure 6A). Interestingly, a completely different scenario was observed after trastuzumab therapy (Figure 6B). A strong 4–5-fold increase in p-Akt was observed 1–6 h after administration of trastuzumab. Later, 1–7 days after onset of trastuzumab therapy, p-Akt levels decreased again, but still remained about 2–3-fold above levels of untreated tumours. Both, ATc and trastuzumab caused a strong increase in Akt1 and Akt2 mRNA expression (Figure 6C and D). However, the kinetics were different in ATc- and trastuzumab-treated mice. Following ATc-mediated ERBB2 downregulation a slow but sustained increase in Akt mRNA expression was observed over the entire exposure period of 7 days (Figure 6C). In contrast, trastuzumab induced a sharp increase in Akt mRNA expression already 1 h after administration, followed by a plateau with levels about 3–4-fold above controls. In a previous study we have already shown a strong dephosphorylation of Erk 1/2 as a consequence of ATc-mediated ERBB2 downregulation (Schiffer *et al*, 2003). In contrast to ATc, trastuzumab did not decrease levels of phosphorylated Erk1/2 as well as total Erk (Figure 7).

### Tumour remission in relation to proliferation and apoptosis

**Figure 8 fig8:**
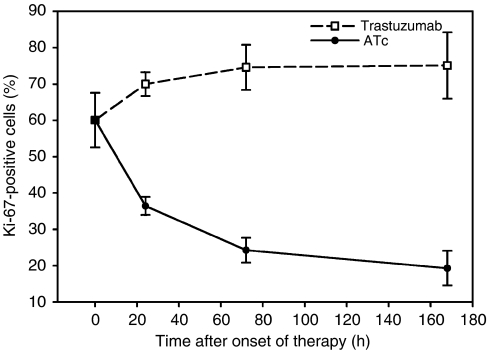
Influence of anhydrotetracycline (ATc) and trastuzumab on proliferation, as evidenced by the fraction of Ki 67-positive tumour cells. Data are mean values and s.e. of 3–5 mice per group.

The fraction of Ki-67 positive nuclei was determined in tumour tissue to study a possible influence of ATc and trastuzumab on proliferation. Downregulation of ERBB2 by ATc led to a strong decrease in proliferating tumour cells (Figure 8). In contrast, trastuzumab did not decrease the fraction of Ki-67-positive nuclei. Therefore, the difference in tumour development after administration of ATc or trastuzumab is reflected by the different fractions of proliferating tumour cells.

Apoptosis as evidenced by the ratio of cytosolic to mitochondrial cytochrome *c* showed a moderate size increase 7 h after ATc administration (Table 1) similar to results obtained in a previous study. Trastuzumab increased apoptosis 24 h after administration (Table 1). However, a decrease was observed after 72 h. In conclusion, apoptosis (Table 1) as well as proliferation (Figure 8) correspond to the differences in tumour development (Figure 5) observed after administration of ATc and trastuzumab.

## DISCUSSION

The emergence of trastuzumab has drastically changed the therapy of breast cancer. Besides improvements in the therapy it led to the concept that only a subgroup of tumours is ‘ERBB2 dependent’ (Weinstein, 2002; Baselga, 2006; Hengstler *et al*, 2006). This underlines the need to direct trastuzumab only to this subset of tumours whose growth depends on ERBB2 signalling. Trastuzumab in tumours not (over)expressing ERBB2 is known to be useless. On the other hand, not all breast carcinomas overexpressing ERBB2 respond to trastuzumab. Although the probability of remission is much better in Herceptest 3+ breast carcinomas compared with tumours with lower levels of ERBB2, still about 50% of Herceptest 3+ breast carcinomas are resistant to trastuzumab. This may be explained by the fact that despite overexpression of ERBB2, tumour growth may predominantly depend on other oncogenes. Therefore, the ideal tumour for trastuzumab therapy should not only overexpress but also be ‘ERBB2 dependent’ (Baselga, 2006). To our knowledge this concept has not yet been challenged, probably due to the fact that it is relatively difficult to determine to which degree tumour growth depends on a particular oncogene.

We used a mouse tumour model where growth of the primary tumours is 100% ERBB2 dependent. ERBB2 mRNA and protein expression can efficiently be downregulated by administration of anhydrotetracycline (ATc) to tumour-bearing mice. Downregulation of ERBB2 leads to a rapid and macroscopically complete tumour remission within 14 days. Tumour remission is accompanied by dephosphorylation of ERK1/2 and AKT/PKB, a strong decrease of Ki-67-positive nuclei in tumour tissue and a moderate increase in apoptosis as evidenced by the cytoplasmic fraction of cytochrome *c*. These data demonstrate a strong ERBB2 dependence of the tumours in our mouse model. Therefore, we initially expected a high efficiency of trastuzumab. Surprisingly, trastuzumab caused only a very weak delay in tumour growth, which is in sharp contrast to the excellent efficiency of Atc-mediated ERBB2 downregulation. We used a route of administration and similar (or even higher) doses of trastuzumab that have been shown to be efficient in previous studies (Baselga *et al*, 1996; Cobleigh *et al*, 1999; Slamon *et al*, 2001). The interaction of trastuzumab with ERBB2 in our mouse tumour model is illustrated by a strong decrease in membrane localisation within 6 h after administration of trastuzumab. However, this did not lead to tumour remission. In agreement with the very moderate influence on tumour growth only a slight decrease in Ki 67 and a very weak increase in cytoplasmic cytochrome *c* were observed. Recently, strong AKT/PKB signalling has been shown to contribute to trastuzumab resistance (Clark *et al*, 2002; Yakes *et al*, 2002; Tokunaga *et al*, 2006). Therefore, we analysed the influence of trastuzumab on the levels of AKT/PKB phosphorylation. Interestingly, trastuzumab caused a sharp 4–5-fold increase in phosphorylated AKT/PKB already within 60–180 min after administration of trastuzumab (Figure 6B). Downregulation of ERBB2 by ATc did not cause such a sharp increase but a moderate-size (1.8-fold) rise in AKT/PKB phosphorylation not before 24 h. Another response to trastuzumab were increased AKT1 and AKT2 mRNA levels (3.5- and 5.3-fold, respectively) already 60 min after administration (Figure 6D) leading to increased levels of AKT-protein (Figure 6B). The rapid phosphorylation of AKT and the increase in AKT RNA and protein may contribute to resistance against trastuzumab.

Recently, we have shown that dephosphorylation of ERK1/2 is relevant for tumour remission after ERBB2-blocking therapy (Hausherr *et al*, 2006). We have shown a strong dephosphorylation of ERK1/2 after Atc-mediated downregulation of ERBB2 using the same mouse tumour model as in the present work (Hausherr *et al*, 2006). In contrast to ATc, trastuzumab did not cause dephosphorylation of ERK1/2. Therefore, the lack in tumour remission after trastuzumab administration may be explained by (i) increased AKT/PKB phosphorylation, (ii) a rapid increase in AKT/PKB mRNA and protein synthesis and (iii) a lack of ERK1/2 dephosphorylation.

**Figure 4:**
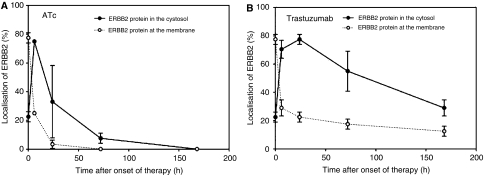
Influence of anhydrotetracycline (ATc) (**A**) and trastuzumab (**B**) on localisation of ERBB2. Both, anhydrotetracycline (ATc) and trastuzumab caused a loss of ERBB2 membrane localisation. Data are mean values and s.e. of 3–5 mice per group.

In our study, a concentration of 40 mg kg^−1^ trastuzumab was used for treatment of mice. To exclude a possible underdosing we analysed membrane localisation of ERBB2. We observed a strong decrease in ERBB2 membrane localisation during the first 24 h after Herceptin administration (Figures 3 and 4). Therefore, the weak response of the tumour does not seem to be a result of underdosing. It should be considered that 40 mg kg^−1^ is a very high dose compared to doses used in other published studies. For instance, Wang *et al* used 0.3 mg kg^−1^ and Scotti *et al* only 200 *μ*g Herceptin per mouse. In both studies Herceptin clearly reduced tumour growth in nude mice (Wang *et al*, 2005; Scotti *et al*, 2007). It should also be considered that human ERBB2 was expressed in our mouse tumour model (Schiffer *et al*, 2003), since Herceptin is directed against the human oncogene. [Fig fig5] [Fig fig6]

The present results show that ‘ERBB2 dependence’ does not automatically include ‘trastuzumab sensitivity’. It will be interesting to analyse whether similar observations can be made in human tumour cells, whereby analysis of functional ‘ERBB2 dependence’ will be more difficult compared to the NIH3T3-HER2 model. Another feature of our mouse model is the compromised immune system, since tumours were induced in nude mice. Therefore, antibody-mediated cytotoxicity due to the immune system will not be observed in the NIH3T3-HER2 mouse model.[Fig fig7] [Fig fig8]

In conclusion, we have demonstrated that ‘ERBB2 dependence’ does not necessarily include sensitivity to trastuzumab, even if cells overexpress ERBB2 and if trastuzumab induces internalisation of the ERBB2 receptor.

## Figures and Tables

**Table 1:** Time after onset of therapy

	**0 h**	**24 h**	**72 h**
ATc	0.05±0.02	0.07±0.03	0.20±0.06^*^
Trastuzumab	0.05±0.02	0.20±0.08^*^	0.11±0.03^*^

Influence of anhydrotetracycline (ATc) and trastuzumab on apoptosis in tumour tissue as evidenced by the ratio of cytoplasmic and mitochondrial cytochrome *c*. Data are mean values and s.d. of three mice per group.

^*^P<0.05 compared to controls.
